# Strategies and Methods for Upscaling Perovskite Solar Cell Fabrication from Lab-Scale to Commercial-Area Fabrication

**DOI:** 10.3390/molecules30102221

**Published:** 2025-05-20

**Authors:** Mengna Sun, Zhiqiang Jiao, Peng Wang, Xiaohu Li, Guangcai Yuan

**Affiliations:** BOE Technology Group Co., Ltd., Beijing 100176, China; sunmengna@boe.com.cn (M.S.);

**Keywords:** perovskite solar cell, large-area fabrication, power conversion efficiency, nucleation

## Abstract

Perovskite, as a promising candidate for the next generation of photovoltaic materials, has attracted extensive attention. To date, the power conversion efficiency (PCE) of perovskite solar cells (PSCs) has reached 26.7%, which is competitive with that of commercial silicon cells. However, high PCE is usually achieved in devices with a small surface area fabricated by the spin-coating method. Perovskite thin films, as the most important layer, suffer from poor uniformity and crystallization caused by the large-area fabrication process, which leads to a dramatic drop in efficiency and exhibits poor reproducibility. Here, we summarize common architectures of PSC and perovskite solar modules (PSMs), as well as analyzing the reasons for efficiency loss on the modules. Subsequently, the review describes the mechanism of perovskite growth in detail, and then sums up recent research on small-to-large-area perovskite devices. Large-area fabrication methods mainly include blade coating, slot-die coating, spray-coating, inkjet printing, and screen printing. Moreover, we compare the advantages and disadvantages of each method and their corresponding mechanisms and research progress. The review aims to provide potential logical conclusions and directions for the commercial large-area perovskite fabrication process.

## 1. Introduction

**Figure 1:**
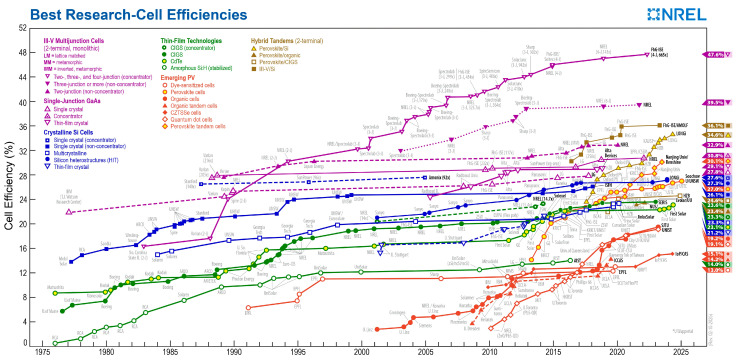
A timeline diagram reported by the National Renewable Energy Laboratory (NREL).

Hybrid organic–inorganic perovskite solar cells (PSCs) have received significant attention for their application in solar cells [1,2,3,4,5], light-emitting diodes [6,7,8,9], laser and photodetectors [10,11,12], etc., due to their excellent advantages of an adjustable and tunable band gap, a large absorption coefficient, a long carrier diffusion length, defect tolerance, a simple structure and fabrication process, etc. Among their various applications, PSCs have been recognized as the best candidate for next-generation optoelectronic devices. Many attempts have been made to improve their performance, leading to an improvement in their power conversion efficiency from 3.8% to a certified 26.7% through the solution fabrication process. This has been achieved through perovskite composition engineering, additive engineering, solvent engineering, and interface engineering strategies [13,14,15,16,17,18,19,20,21]. High power conversion efficiency is common in small-area devices (≤1 cm^2^) [22,23,24]. A timeline diagram of the best research-cell efficiencies reported by the National Renewable Energy Laboratory (NREL) is shown in Figure 1.

When upscaling solar cells from laboratory to commercial levels, PCE reduction frequently occurs. This results primarily from inadequate interfaces between the perovskite and transport layers, including the electron transport layer (ETL) and hole transport layer (HTL), and the quality of perovskite thin films [25,26,27,28]. The quality of the perovskite thin film is a key issue, since producing a good quality of thin film over a large area is difficult to achieve. This is because the uniformity and crystallization of the whole film is difficult to control, which has a critical influence on the performance of large-area devices. Therefore, fabricating a uniform perovskite film with good morphology and crystallization has become an essential part of achieving a high-performance and easily reproducible perovskite device, which is vital for the commercial large-area fabrication process [20,29,30,31]. According to the literature, large-area fabrication methods mainly include blade coating, slot-die coating, spray-coating, inkjet printing, screen printing, etc. To date, these methods have achieved efficiencies of approximately 20% [32,33,34,35,36,37]. Notable PCEs of 25.31% and 23.34% based on a-phase FAPbI_3_ solar cells for small-area cells (0.09 cm^2^) and mini-modules were obtained using the meniscus-modulated blade coating method combined with solvent engineering. PbI_2_ redistribution can enable in situ passivation for blading inverted PSCs with 24.5% and 20.4% efficiency for small-area PSCs and PSCs with an area of 13.68 cm^2^, respectively [38,39]. With the slot-die coating method, large-area modules (17 cm^2^) achieved power conversion efficiencies of 20.4%, and 156 × 156 mm^2^ perovskite solar modules exhibited a PCE of 19.7% [40,41] Overall, blade coating and slot-die coating are the best methods for achieving higher efficiency.

Here, we systematically summarize common architectures of PSCs and PSMs. We subsequently describe the mechanism of perovskite growth in detail, and then sum up the recent research on PSCs, from small-area to large-area PSCs. Finally, we discuss the best method for scaling up from lab-scale to commercial-area fabrication, as well as comparing the advantages and disadvantages of each method and their corresponding mechanisms and research progress. The review aims to provide potential and logical conclusions and directions for the commercial large-area perovskite fabrication process.

## 2. Architecture of Perovskite Devices

### 2.1. Architecture of PSCs

**Figure 2:**
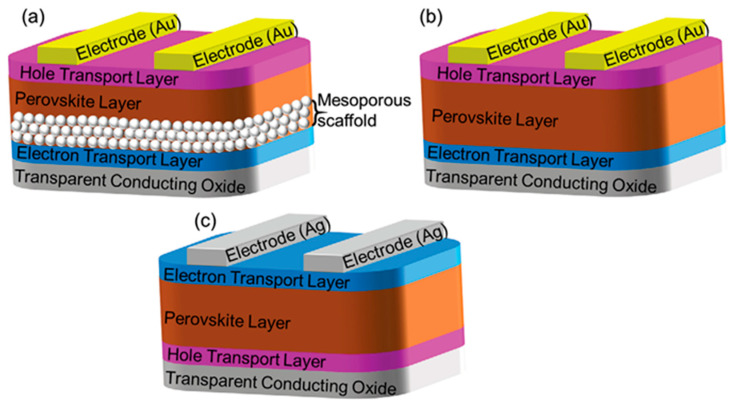
Schematic architecture of (**a**) mesoporous PSC and (**b**,**c**) regular and inverted planar PSCs. Reproduced with permission [42].

As depicted in Figure 2, the common typical architectures of PSCs can be summarized into two types: the mesoporous structure and the planar heterojunction structure [42].

For the mesoporous structure, a light-harvest absorber thin film is usually deposited on the scaffold with a compact capping layer. The mesoporous layer can help to facilitate the efficiency of the charge separation to achieve high PCE, along with a reduced hysteresis effect. On the other hand, for the planar structure, there is no mesoporous scaffold in the architecture, and a perovskite thin film is commonly deposited on the compact ETL or HTL. Furthermore, according to the position of the HTL and ETL, the structure can be divided into a regular n-i-p structure and an inverted p-i-n structure. The simplified working mechanism of PSCs is thought to involve the perovskite absorber layer capturing light, and then generating electron–hole pairs. Since the exciton binding energy is very low, it is easy for excitons to be dissociated to produce free electron–hole pairs. As a result, the pairs can be collected by electron and hole contacts, respectively.

### 2.2. Architecture of PSMs

**Figure 3:**
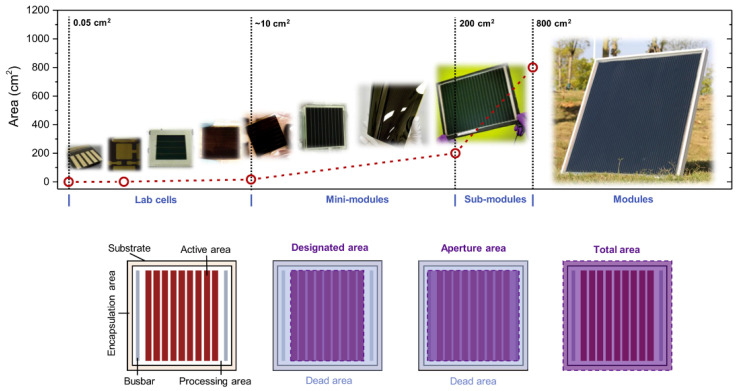
Classification of PSC modules by size and definition of areas used for PSC module performance measurement. Reproduced with permission [43].

As shown in Figure 3, PSMs can be classified into four categories, according to their size: mini-modules (<200 cm^2^), sub-modules (200–800 cm^2^), small modules (800–6500 cm^2^), standard modules (6500–14,000 cm^2^), and large modules (>14,000 cm^2^). Furthermore, the structure of a PSM can be divided into an active area and a dead area. Among these, the active area is defined as the overlap area of every functional layer, and plays a role in light absorption and power conversion; and the dead area, calculated based on the interconnection area, does not generate any electricity.

The PCE of large-area PSCs can be significantly reduced as a result of a large amount of resistance loss in the transparent conducting electrode, induced by a long transport distance. In order to solve this problem, it is necessary to divide the large-area solar cell into small sub-cells, and form an interconnected cell module through series interconnection.

**Figure 4:**
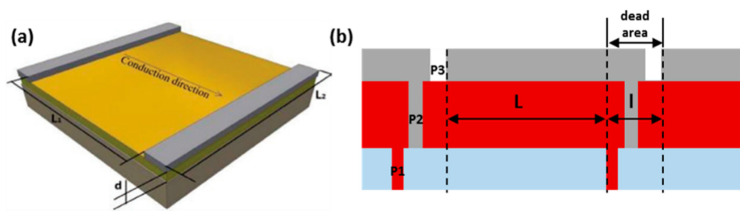
(**a**) Electrical losses caused by resistance and (**b**) schematic illustration of series-connected perovskite module. Reproduced with permission [44].

As demonstrated in Figure 4, the design of the series connection can sum up the voltages of single sub-cells, while the current of the module is same as that for each single sub-cell. In the fabrication of the solar cell module, it is important to divide the cell by laser or mechanical scribing methods, including three processes (P1, P2, and P3). From bottom to top, P1 is used to separate the bottom electrode, which is commonly made from ITO or FTO. Then, P2 is carried out to remove the organic or inorganic materials and form a channel for the interconnection of the single sub-cells. Finally, P3 is carried out to split the top metal electrode and the boundary. The relationship between the PCE of the cells and that of the module can be defined according to the following formula:

PCE_module_ = PCE_cell_ GFF

Meanwhile, the geometric fill factor (GFF) is calculated using the following equation:

$$\mathrm{GFF}=\frac{\;\mathrm{Active}\;\mathrm{area}}{\mathrm{Total}\;\mathrm{area}}=\frac{n\times w\times L}{n\times w\times \;(L+l)}=\frac{L}{L+l}$$

In the above formula, n represents the number of single cells, w represents the width of single cells, and L and l are the lengths of the active part of the single cells and the interconnected part (patterning part) between single cells, respectively.

In addition to the electrical losses caused by the resistance, the geometric losses ascribed to the dead area and the corresponding optical losses from reflection and absorption, etc., can also induce a reduction in the PCE [44].

## 3. Nucleation Process of Perovskite

**Figure 5:**
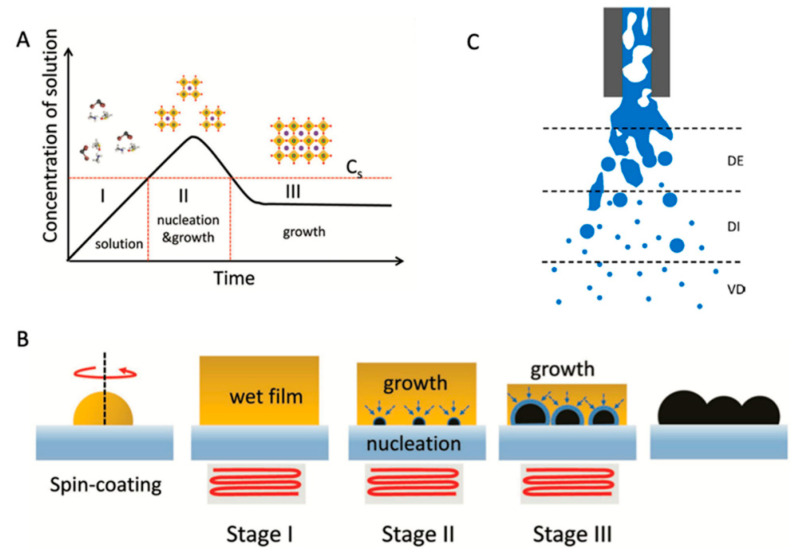
(**A**) A Lar Mer model for the nucleation and growth of perovskite thin films. (**B**) A schematic illustration of the nucleation and growth of perovskite films at each stage. (**C**) A schematic representation of the three flow regimes, based on spraying for fuel combustion, during atomization: dense (DE), dilute (DI), and very dilute (VD) with respect to the concentration of the liquid in the spray cone. Reproduced with permission [46].

As we know, the quality of perovskite, such as its uniformity and crystallization, plays an important role in ensuring a high PCE of perovskite devices,. The mechanism of perovskite growth was investigated to obtain the films with good quality [45,46,47], as shown in Figure 5. According to classical nucleation theory, this process mainly consists of three stages: (i) As the solvent evaporates, the formation of perovskite nuclei appears when the solution concentration reaches a supersaturation concentration (Cs represents the supersaturation concentration of a precursor solution); (ii) Because of the interaction between the solute and the solvent, the growth enters a diffusion-limited regime, and nucleation and growth occur simultaneously in this stage; (iii) With a drop in solute concentration, new nuclei formation mainly occurs in the final stage [46,48]. The key parameters for high-quality perovskite thin films are recognized as an increase in the amount of nuclei and slowing down of the growth process through supersaturation control by removal of host solvents or the assistance of chemical additives, etc. [46,49,50].

## 4. Fabrication of Perovskite Devices, from Small- to Large-Area Fabrication

**Figure 6:**
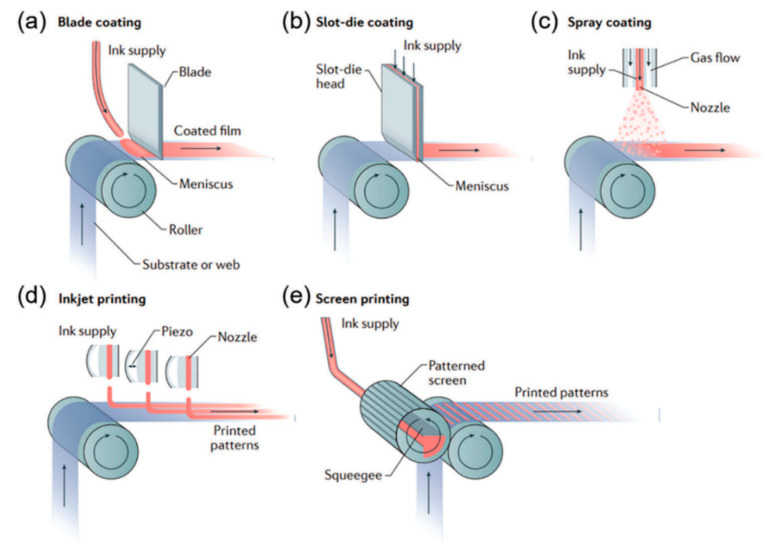
Schematics of production processes using some typical techniques. (**a**) Blade coating; (**b**) slot-die coating; (**c**) spray-coating; (**d**) inkjet printing; (**e**) screen printing. Reproduced with permission [51].

As we know, perovskite thin films are a key layer, and many attempts have been made to develop the depositing method in order to achieve better performance. Spin-coating is usually carried out to achieve a good-quality film in small-area fabrication. Blade coating, slot-die coating, spray-coating, inkjet printing, screen printing, etc., are more suitable for large-area fabrication [51], as depicted in Figure 6.

### 4.1. Spin-Coating

**Figure 7:**
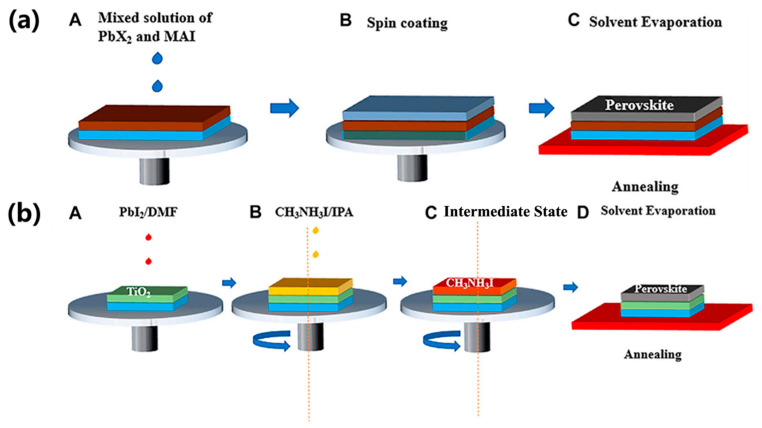
A schematic diagram for the process of obtaining a perovskite layer by the one- or two-step spin-coating method. (**a**) One-step method and (**b**) two-step method. Reproduced with permission [52].

The spin-coating method is widely used for the fabrication of thin films over areas of up to 4 inches. As shown in Figure 7, there are two common spin-coating methods to obtain perovskite thin films, usually named the one-step and the two-step method. The two methods allow for the achievement of high-performance small-area cells through dipping the substrate into a small amount of perovskite precursor, and then rotating to achieve a good-quality thin film [52].

Recently, some modified methods based on these two methods have also been developed for better fabrication of perovskite films with good morphology and crystallization. In 2014, Xiao, M., et al. [53] reported a fast deposition–crystallization procedure (anti-solvent engineering) for highly efficient solar cells. The introduction of chlorobenzene or toluene as an anti-solvent to control perovskite crystal growth led to smooth and high-quality films. The resulting planar solar cells yielded an average PCE of 13.9 ± 0.7 % and a steady-state efficiency of 13%. Then, in 2015, Yang, W.S., et al. [54] reported an approach for achieving high-quality and well-crystallized FAPbI_3_ films by the direct intramolecular exchange of DMSO intercalated in PbI_2_ with FAI. The approach can produce FAPbI_3_ films with a favorable crystallographic orientation, along with large-grained dense microstructures and smooth surfaces. As a result, FAPbI_3_-based PSCs showed a PCE of over 20%. In 2016, Guo, Y., et al. [55] exhibited that introducing NH_3_SO_3_ as an additive could improve the performance of solar cells by controlling the morphology and crystallinity of the perovskite film, resulting in the enhancement of the PCE from 13.08% to 16.02%, and devices without encapsulation demonstrated good long-term stability. In 2017, Sun, M., et al. [1] reported that a solution-processed CH_3_NH_3_PbI_3_-based device using dopant-free Me-QTPA achieved a PCE of 9.07%. Dopant-free PSCs with Me-QTPA showed better performance than dopant-free spiro-OMeTAD, especially in terms of long-term stability; in the same year [4], a rapid and simple process to prepare the PSCs in ambient air by adding 2-pyridylthiourea to the precursor solution was also demonstrated. The PSCs with added 2-pyridylthiourea exhibited a PCE of 18.2%, showing an 18% increase and less hysteresis compared with the cells without additives. From 2015 to 2019, the PCE increased rather slowly, but continuously, and eventually reached 25.2% [56,57,58]. In addition to cation-doping strategies, substitution of X-site halide anions can also significantly affect the optoelectronic properties of FAPbI_3_ perovskites. In 2021, Jeong, J., et al. [59] introduced HCOO- to fill the halide vacancy defects, facilitating the enhancement of the crystallinity of FAPbI_3_. With doping of 2% concentration, the larger grain size achieved, together with the better crystal orientation, was better for carrier transport, and could efficiently suppress the non-photoactive δ-FAPbI_3_ phase. Moreover, the FAPbI_3_-based PSCs with pseudo-halide treatment attained a record PCE of 25.6% (certified 25.2%) and a VOC of 1.19 V. In 2023, Liang, Z., et al. [14] reported that for FA_1−x_CsxPbI_3_-based solar cells, incorporation of Cs cations can enable the formation of a perovskite lattice, but compositional inhomogeneity segregation is more likely to be harmful to the performance of the solar cells. They devised a strategy using PSP to homogenize the cation components in the perovskite films. The resultant p-i-n devices yielded a certified steady-state PCE of 25.2% and durable stability. In 2024, Zhou, J., et al. [22] exhibited a novel HTM (T2) to achieve better performance. T2 exhibits strong interactions with adjacent layers, resulting in efficient inhibition of interlayer ion migration. A PCE of 26.41% (certified 26.21%) and a certified PCE of 24.88% were demonstrated for areas of 0.1 and 1.0 cm^2^. Its excellent performance, together with its scalable and low-cost synthesis, provides it a potential application in future large-scale PSCs. However, even though fabrication with the spin-coating method achieves the most highly efficient PSCs, this method is not suitable for large-scale fabrication processes, due to the radial non-uniformity caused by centrifugal force [60]. Spin-coating is the most commonly used method in the laboratory; it is more suitable for small-area fabrication, and has the advantages of good uniformity of the films and excellent repeatability. However, its disadvantages are that it is difficult to enlarge the process, since the large-area uniformity achieved with this method is poor and the edge effect is obvious. In addition, the upscaling of this method of fabrication is also limited by its low material utilization rate and the slow speed of the process.

### 4.2. Blade Coating

**Figure 8:**
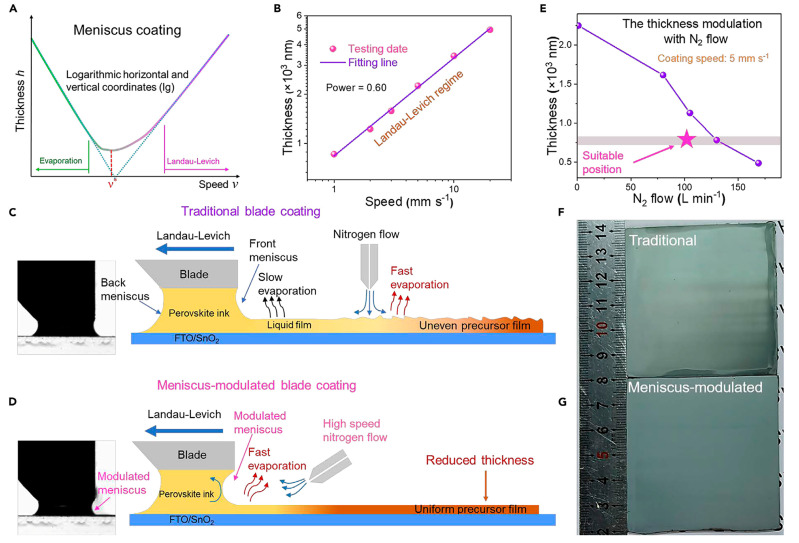
Comparison of traditional and meniscus-modulated blade coating. (**A**) Film thickness as a function of coating speed. Two deposition regimes are identified: evaporation and Landau–Levich. (**B**) Deposition regime measured in this work. (**C**,**D**) Schematic diagram of traditional and (**D**) meniscus-modulated blade coating. (**E**) Relationship between thickness of perovskite films prepared by meniscus-modulated blade coating with nitrogen flow. (**F**,**G**) FAPbI_3_ films prepared by (**F**) traditional blade coating and (**G**) meniscus-modulated blade coating. Reproduced with permission [39].

Blade coating is also a widely used technology in large-scale fabrication processes, and has advantages in that it is a high-throughput and scalable process. During the process, ink is first spread onto the substrate, followed by the generation of a uniform wet film through the movement of the blade coater. Achieving high-performance cells or modules requires precise control of the layer thickness and homogeneity of thin films. There have been many studies reporting on the fabrication of scalable perovskite films using blade coating. In 2015, Yang, Z., et al. [61] reported that in cells with the structure of ITO/PEDOT:PSS/CH_3_NH_3_PbI_X_Cl_3−X_/PC_61_BM/Bis-C_60_/Ag, high-quality interlayer films could be obtained by optimization of the blade coating process and relative humidity conditions, and a high PCE of 10.44% of the device could be achieved. In 2017, Li, C., et al. [62] embedded the surfactant-like monoammonium zinc porphyrin (ZnP) compound into perovskite film to obtain large-area uniform perovskite films as large as 16 cm^2^ by the blade coating method. Meanwhile, for areas of 1.96 cm^2^ and 0.1 cm^2^, an efficiency of 18.3% and 20.5% could be achieved, respectively. Tang, S., et al. [63] achieved purer-phase perovskite thin films by incorporating a small amount of cesium and bromine ions into the precursor solution. In addition, perovskite thin films with micrometer-sized grains and without pin-hole could be obtained through the introduction of methylammonium chloride. The best device fabricated with doctor-bladed MA_0.6_FA_0.38_Cs_0.02_PbI_2.975_Br_0.025_ films achieved a PCE of 19.3%, and was able to retain 90% of its initial PCE after 30 days. In 2018, Li, J., et al. [64] identified that including the intermediate phases in the phase transition is a key issue in rationally transitioning from the spin-coating to the blade coating process. The study reported that a dense perovskite film with a large-grained size and excellent crystal quality could be obtained through direct crystallization. As a result, the blade coating-fabricated planar cell demonstrated photovoltaic properties with efficiencies of 18.74% (0.09 cm^2^) and 17.06% (1 cm^2^). In 2020, Huang, S.-H., et al. [65] presented the fabrication of highly efficient PSCs by using the blade coating method with a solvent mixture of γ-butyrolactone and dimethyl sulfoxide in an ambient environment. By regulating the influencing factors, such as the interface, morphology, and crystallinity of perovskite films, by tuning compositional variations and the introduction of additives, a high power conversion efficiency of 17.02% was achieved in air. Recently, notable PCEs of 25.31% and 23.34%, based on a-phase FAPbI_3_ solar cells for small-area cells (0.09 cm^2^) and mini-modules (a certified PCE of 23.09%), were reported by Huang, C., et al. [39], who used a meniscus-modulated blade coating method combined with solvent engineering, as shown in Figure 8. In addition, this mini-module could retain over 93% of its initial PCE after aging for 2000 h outdoors.

In addition, Zhao, X., et al. [66] developed a scalable vapor-phase fluoride treatment to facilitate a uniform and stable perovskite surface, resulting in the suppression of defect formation. The study achieved PCEs of 24.8% for 0.16 cm^2^ single cells and 18.1% for 228 cm^2^ solar modules, with T_80_ lifetimes of 43,000 ± 9000 h under standard sun illumination at 30 °C. Blade coating is a convenient and compatible method, with the advantages of low fabrication cost and an easy-to-control process. It shows potential as a method for the large-scale fabrication of perovskite films. However, there are still many operating conditions that need to be further optimized for compact, pinhole-free, uniform, and well-crystallized perovskite films. Blade coating is characterized by great extensibility and a high material utilization rate (90%). In addition, its demand for equipment is low, resulting in a low cost of production. The above factors determine that blade coating is more suitable for large-area fabrication. However, the blade coating method is more sensitive to process parameters, such as coating speed or pressure, etc., which leads to difficulty in crystallization control, accompanied by pinholes and uneven thickness, etc.

### 4.3. Slot-Die Coating

Slot-die coating is similar to the blade coating method, but it demonstrates the advantages of a higher yield and reproducibility, resulting from precise control via a machined microfluidic die. By tuning the temperature and quenching process, along with using an adjustable precursor ink, this is a competitive technique for use in the large-area fabrication process. Fievez, M., et al. [67] applied a synergistic crystallization strategy, along with gas quenching and substrate heating, to promote perovskite crystallization formed through slot-die coating of Cs_0.16_FA_0.84_Pb(I_0.88_Br_0.12_)^3^ on 10 × 10 cm^2^ substrates. As a result, MA-free PSCs could reach an impressive PCE of 18% on a 0.09 cm^2^ active area. This study shows the potential of using this slot-die technology for the controlled coating and homogeneous crystallization of perovskite films over large-area substrates (~10 × 10 cm^2^). In 2020, Du, M., et al. [68] used a high-pressure nitrogen extraction (HPNE) strategy to facilitate better crystallization during the slot-die coating process, forming a wide window for producing perovskite thin films of good quality. HPNE can help to generate the crucial intermediate phase, which is crucial to obtain excellent performance of the cells. This strategy provides a novel direction for large-area device fabrication, resulting in a PCE of 19.4% in a 40 × 40 mm^2^ module.

**Figure 9:**
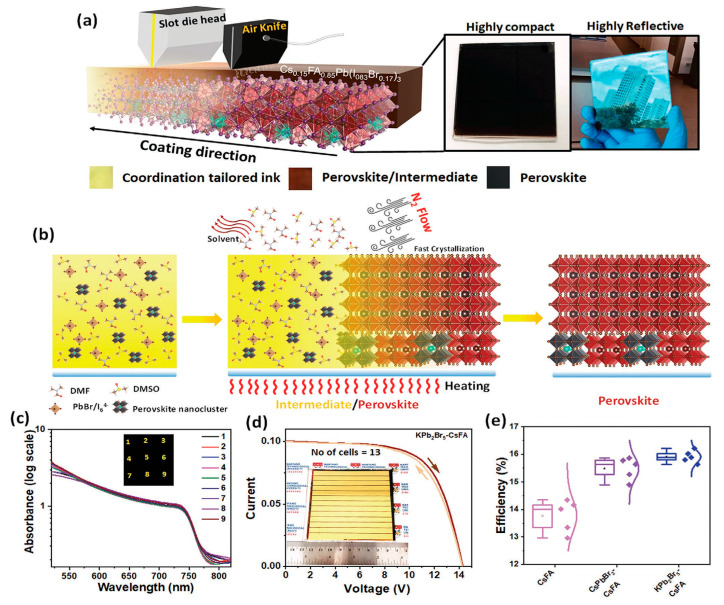
Coordination-tailored perovskite inks for slot-die coating of perovskite films. (**a**) Schematic presentation of N_2_-knife-assisted slot-die coating of perovskite films at 10.8 mm s^−1^ and 56 °C using coordination-tailored ink. Insets show pictures of as-coated perovskite ink, perovskite/intermediate film, and perovskite film. (**b**) Schematic representation of drying of perovskite ink, followed by perovskite/intermediate film and completely crystallized perovskite film. (**c**) UV-vis absorption at 9 different areas on 10 × 10 cm^2^ glass/perovskite substrate. (**d**) J–V curve of best-performing KPb_2_Br_5_-CsFA perovskite solar module with series connection of 13 sub-cells with an active area of 57.5 cm^2^ (inset: perovskite solar module). (**e**) Device performance statistics for CsFA, CsPbBr_3_-CsFA, and KPb_2_Br_5_-CsFA PSC modules. Reproduced with permission [69].

In 2021, as shown in Figure 9, Rana, P.J.S., et al. [69] presented a seed-assisted crystallization approach, with the addition of the alkali salts CsPbBr_3_ and KPb_2_Br_5_ to the perovskite precursor ink, which enabled the formation of homogeneous and more favorably crystalline Cs_0.15_FA_0.85_Pb(I_0.83_Br_0.17_)_3_ perovskite thin films via a slot-die coating technique. The corresponding perovskite module of 57.5 cm^2^ demonstrated an efficiency of 16.22% and kept 82% of its initial PCE after 4800 h at 30% relative humidity without encapsulation. Zimmermann, I., et al. [70] developed a sequential deposition method to make a triple cation perovskite by slot-die coating. A mixture of lead iodide and cesium iodide was firstly deposited onto the substrate, followed by deposition of organic cations to form perovskite. After optimization of the ink composition and deposition parameters, a PCE of 19% and 15.2% was achieved for small solar cells and 12 cm^2^ mini-modules. In 2023, Li, J., et al. [71] reported that ribbing effects exist in slot-die coating, but adjusting the precursor ink’s rheological properties can sufficiently solve this problem. The viscosity was adjusted by the introduction of acetonitrile to FAPbI_3_ precursor inks based on 2-methoxyethanol. With an ACN co-solvent content of 46 vol%, a mini-module of 12.7 cm^2^ exhibited a PCE of 17.1%; furthermore, the PCE was maintained at close to 100% after placing the module outdoors in winter and leaving it there for 1 year. Since filling the ink reservoir and supply pipe requires an increased amount of solution during the process, and since there are fewer reported instances of its utilization, this technology needs more time and attempts to develop it further. Similarly to blade coating, slot-die coating is also sensitive to process parameters. However, it demonstrates better performance in terms of its material utilization rate (95%) and film uniformity, meaning that it is suitable for continuous production, such as in roll-to-roll processes. More importantly, this method requires larger quantities of precursor ink to fill the reservoir and pipe, which means that it is not suitable when a frequent supply of precursor ink is not possible. Therefore, slot-die coating has been less investigated, and modules fabricated with this method demonstrate a lower PCE than those obtained by blade coating.

### 4.4. Spray-Coating

**Figure 10:**
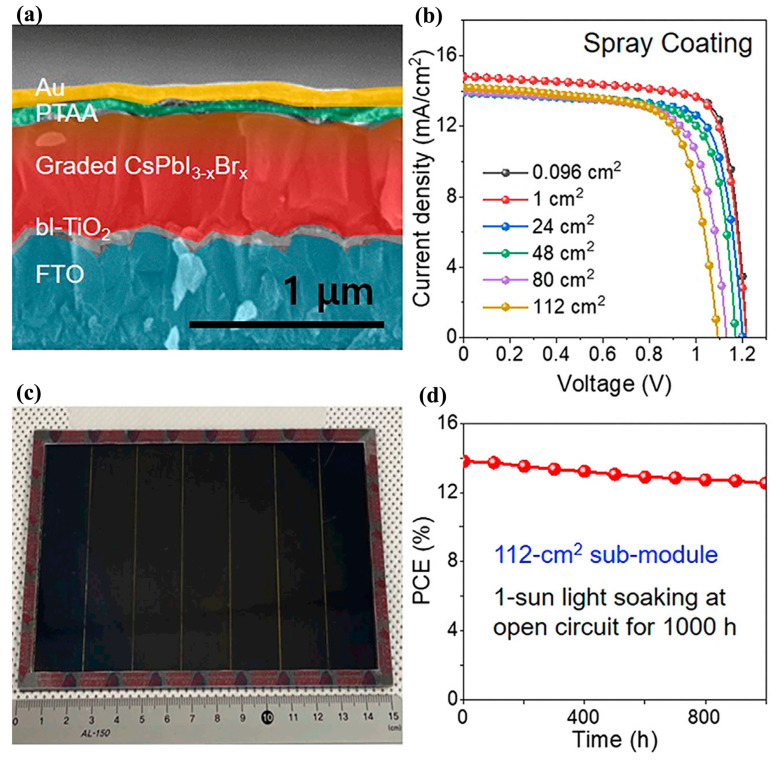
(**a**) Cross section of device; (**b**) PCE of devices with different areas; (**c**) view of perovskite module; (**d**) stability of module under open-circuit sunlight soaking for 1000 h. Reproduced with permission [76].

Spay-coating is a common industry deposition method to fabricate large-area thin films; it includes spray printing, pyrolysis, and deposition, as well as ultrasonic spray. This technique produces more homogeneous thin films over large areas. Research on defect-less large-area perovskite films is the main focus in the development and optimization of this technology. In 2014, Barrows, A.T., et al. [72] first investigated processing parameters using the spray-coating technique, focusing on the influence of the temperature of the substrate, the solvent volatility, and annealing on the performance of solar cells. The study confirmed that the maximum PCE was correlated with dense films with a surface coverage above 85%. With this approach, cells with a PCE of 11% could be obtained. In 2018, Chou, L.-H., et al. [73] reported a scalable ultrasonic spray method to fabricate perovskite thin films by precisely controlling the concentration of the precursor solution and spray passes over a 1 cm^2^ active area. PCEs of up to 12.30% were achieved, with less hysteresis. Meanwhile, when the area was scaled up to 2 and 3 cm^2^, PCEs of 10.18% and 7.01% were achieved, respectively. In 2019, Jiang, Y., et al. [74] reported a new deposition method by combining raster ultrasonic spray-coating and chemical vapor deposition to fabricate PSCs without limitations in terms of the coating area. A FAPb(I_0.85_Br_0.15_)_3_-based device exhibited a PCE of 14.7% for an area of 12 cm^2^. Moreover, the device showed good stability, and an average T_80_ lifetime of 535 h and 388 h for small cells and modules, respectively. In 2020, Cai, H., et al. [75] systematically optimized the parameters of perovskite thin films produced using the spay-coating process, including their thickness, anti-solvent bath, and thermal annealing time, which had an influence on the kinetics of crystallization. As a result, an impressive PCE of 20.6% could be obtained. The study demonstrated that spray-coating can be applied to the commercial deposition of scalable solar cells. As demonstrated in Figure 10, Heo, J.H., et al. [76] upscaled CsPbI_2_Br-based perovskite cells from 0.096 cm^2^ to 112 cm^2^, resulting in enhancements in PCE varying from 14.04% to 10.82%. The results confirmed that the PCE decreased by 3% when the area was increased by 1000×. By adjusting the spray time of the precursor solution, an improved composition profile of the resulting graded CsPbI_3-x_Br_x_ surface layer could be obtained, which could broaden the absorption wavelength range and increase the carrier lifetime. The corresponding PCEs of the small cells and modules could be increased to 16.81% and 13.82%.

In 2023, Yu, X., et al. [77] reported a moisture-assisted strategy to eliminate the “coffee-ring” effect that can appear in spray-coating, with the utilization of humidity control and the introduction of water additives. Cs_0.19_FA_0.8_1PbI_2.5_Br_0.5_ was used to make a cell with homogeneous morphology and excellent crystallization, and a PCE of up to 16.75% was achieved for an area of 64.8 cm^2^. Spray-coating, as an emerging technology, faces challenges such as poor crystallization and coverage, as well as non-uniform distribution resulting from dendrite formation and defects on the surface. In addition, it is difficult to accurately control the film thickness within the nm range. The use of this method often results in a low V_OC_, J_SC_, and FF. More research on this method needs to be carried out to solve these problems. Spray-coating has advantages for application to complex shapes as it can cover non-planar substrates, and also has a high material utilization rate. However, droplet splashing can result in a rough film, causing a decrease in the efficiency of devices. This, along with parameter optimization, is a big challenge that needs to be overcome in further development.

### 4.5. Screen Printing

**Figure 11:**
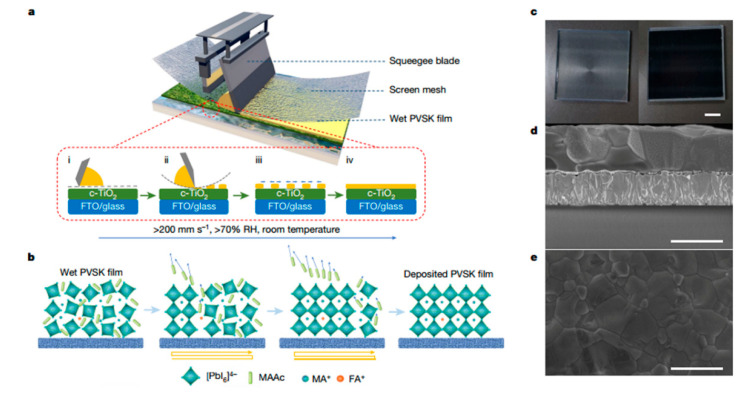
A diagram of the screen printing method for the deposition of perovskite thin films. (**a**) A schematic of the transfer/leveling procedure during the formation of perovskite (PVSK) thin films by means of the screen printing process. (**b**) A schematic of the thermal annealing process. (**c**–**e**) Optical and SEM cross section and surface images of the perovskite thin films. Reproduced with permission [85].

Screen printing is a widely used film deposition technique, whereby a screen is applied to transfer ink onto a flat substrate. Printed patterns can be obtained through the open mesh apertures of the screen. Meanwhile, non-printed areas are delineated by a blocking stencil. This technology has advantages in that it can distribute the print ink uniformly for better permeation of the substrate. However, the stability and tunable viscosity of the precursor ink, which is made from a common organic solvent, restrict the further development of the technology. Many attempts have been made to develop an efficient perovskite ink. In 2009, Kojima, A., et al. [3] first deposited mesoporous TiO_2_ paste onto compact TiO_2_ using the screen printing method, and a PCE of 3.8% was achieved. Following the success of screen printing for PSCs, the corresponding functional layer was further developed for the fabrication of devices including ETLs, HTLs, and electrodes [78,79,80,81,82,83]. In 2014, Mei, A., et al. [84] introduced 5-AVA into the perovskite absorption layer to enhance the crystallinity of MAPbI_3_-based perovskite, as well as the passivation of defects. The fully printed PSC achieved a certified PCE of 12.8%, and was stable for >1000 h in ambient air under full sunlight. In 2018, via registration of the overlapping layers, De Rossi, F., et al. [80] demonstrated the use of screen printing to manufacture A4-size modules with an active area of 198 cm^2^. The un-encapsulated modules exhibited unexpectedly high performance, with a remarkable 6.6% PCE after aging for two months. In 2023, Chen, C., et al. [85] exhibited a stable and viscosity-adjustable perovskite ink made from MAAc, resulting in perovskite thin film thickness control within 120 nm to 1200 nm over an area of 0.5 × 0.5 cm^2^ to 5 × 5 cm^2^, as demonstrated in Figure 11. With printing rates of up to 20 cm s^−1^, PCEs of 20.52% and 18.12% could be achieved for areas of 0.05 and 1 cm^2^. Moreover, the full-printed device with an area of 16.37 cm^2^ obtained an 11.8% PCE, and showed good stability. This method requires pastes or highly viscous ink. However, the development of the screen printing technique is restricted by its cost and the complexity of cleaning the inside of the screen. In 2024, He, J., et al. [86] reported a novel “two-in-one” defect passivation strategy through doping TiO_2_ paste with CsX (X = F, Cl, Br, I) to integrate CsF. F- could rectify oxygen vacancies, and Cs^+^ could efficiently mend methylamine vacancies, resulting in a significant improvement in PCE from 16.18% to 18.24%. This approach offers a potential direction for the enhancement of device performance. Screen-coating is recognized as one of the easiest and lowest-cost methods for the fabrication of PSCs. At the same time, this method has good patterning ability. However, the fabrication of perovskite thin films by screen-coating still remains a challenge, due to the low viscosity and instability of perovskite precursor ink.

### 4.6. Inkjet Printing

**Figure 12:**
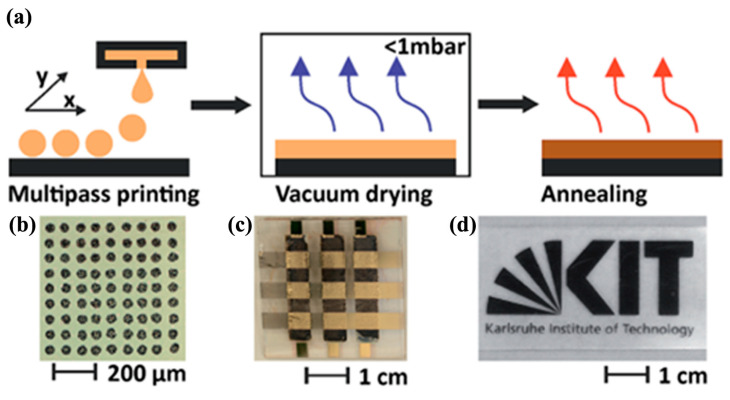
(**a**) Multipass inkjet printing process and (**b**–**d**) optical image of corresponding film and devices. Reproduced with permission [88].

Even though screen printing is suitable for large-area PSC fabrication processes, the high viscosity of the ink makes thickness control very difficult, and there is also the possibility of wastage of a large quantity of ink and many defects during the process. Inkjet printing is a feasible and low-cost method that can be used as an alternative to solve these problems; this includes drop-on-demand (DoD) and continuous inkjet printing. Nozzles are employed to distribute the precursor ink to form a fine and controlled pattern. In 2014, Wei, Z., et al. [87] first demonstrated the fabrication of planar PSCs with a nanocarbon hole-extraction layer by an inkjet printing technique. PbI_2_ could be transformed into CH_3_NH_3_PbI_3_ by an in situ method through designing a carbon plus CH_3_NH_3_I ink. As a result, the PCE could increase to 11.6% with the reduced amount of charge recombination, constituting a major step towards the fabrication of low-cost, large-scale, and highly efficient perovskite devices. In 2016, Mathies, F., et al. [88] reported the fabrication and optimization of multipass inkjet-printed PSCs, as depicted in Figure 12. A homogenous and compact perovskite film with a large crystal size could be obtained through control of the crystallization and thickness of the perovskite thin film and an additional vacuum annealing treatment. The optimized multipass inkjet-printed PSC devices demonstrated power conversion efficiencies of up to 11.3%, while the spin-coated reference devices achieved PCEs of 12.8%, which provides technical support for the further development of inkjet coating methods.

In 2018, Li, P., et al. [89] systematically studied the physical properties of ink solution and its droplet wetting behavior on a mesoporous substrate. Via the use of precisely controlled micro-droplets in inkjet printing, a uniform liquid film was achieved. Mesoporous substrates facilitated fast and complete coalescence of ink droplets, as well as limiting random diffusion. A homogeneous PbI_2_ film was created with the use of compact perovskite with a micro-scale crystal size, resulting in high PCEs of 18.64% for small-area PSCs (0.04 cm^2^) and 17.74% for large-area PSCs (2.02 cm^2^). In 2019, Huckaba, A.J., et al. [90] described the inkjet printing of mesoporous TiO_2_ and perovskite, and the corresponding fabrication of a highly efficient PSC. The printed TiO_2_ showed an open-pore morphology and homogeneous surface coverage ranging from 1 mm^2^ to >10 cm^2^. As a result, an impressive PCE of 18.29% was obtained. Achieving fully IJP PSCs is still a big challenge in further development. In 2020, Schackmar, F., et al. [91] reported on PSCs with an all-IJP ITO/NiOx/perovskite/BCP/Au architecture; the corresponding devices demonstrated an impressive PCE of 17.2%, with low hysteresis. The study showed the detailed parameters for each IJP layer; scalability and freedom in the design of printing patterns was exhibited by the prototype devices. Tan, L., et al. [92] showed that 2-ADAHCl could improve devices with its efficient passivation capability. By drop-on-demand inkjet printing with a quantitative 2-ADAHCl, perovskite surface defects were suppressed, together with the improvement of interfacial contact between perovskite and HTL. As a result, PSCs with a PCE of 24.57% were achieved. Meanwhile, the devices demonstrated improved operational and environmental stabilities. Inkjet printing also has a high-precision pattern ability, which is suitable for special designs. However, during the fabrication process, a high amount of drip is required. The process has a low mass production efficiency due to the restriction of slow speed. At the same time, there are strict requirements for the ink to have a low viscosity and specific rheological properties, etc. All of the above factors need to be improved for further applications. The large-area production of perovskite solar cells by inkjet printing will depend on the printing speed and device structure.

Table 1 summarizes the different large-scale fabrication methods. To make the comparison more clear, the results are for a PSC active area of less than 1 cm^2^ in all cases. Furthermore, in order to improve the depth of the manuscript, Table S1 in the Supplementary Materials provides the detailed parameters of these methods.

## 5. Summary and Perspectives

Perovskite, as a promising candidate for the next-generation production of photovoltaic materials, has attracted increasing attention in the scientific and industrial fields. To date, through perovskite composition engineering, additive engineering, solvent engineering, and interface engineering strategies, etc., the power conversion efficiency of small-area PSCs has achieved a certified 26.7%, which is competitive with that of commercial silicon cells. However, a high PCE is usually achieved in small-area devices fabricated by the spin-coating method. Issues in upscaling fabrication from lab-scale to commercial-area fabrication have become the key point for future industrialization. Among these issues, perovskite thin film, as the most important layer, suffers from poor uniformity and crystallization caused by large-area fabrication, which lead to a dramatic efficiency drop and bad reproducibility. During large-scale fabrication processes, factors including the film fabrication method and quality control need to be considered to achieve high PCE. Here, we summarized common architectures of PSCs and modules, and analyzed the reasons for efficiency loss in these modules. Then, the review described the mechanism of perovskite growth in detail, and then summed up the research on PSCs, from small-area to large-area PSCs, conducted in recent years. Widely used large-area fabrication methods mainly include blade coating, slot-die coating, spray-coating, inkjet printing, screen printing, etc. Moreover, we compared the advantages and disadvantages of each method and their corresponding mechanisms and research progress, respectively. The review aims to provide potential logical conclusions and directions for further development of commercial large-area perovskite fabrication processes. Up until now, the abovementioned methods have been able to achieve an efficiency of ~20%. Meanwhile, the PCE of perovskite–silicon tandems has reached over 30%, surpassing other types of tandems based on perovskites.

However, large-area fabrication still faces challenges that must be solved for further commercial application. To achieve commercialization, more attention needs to be paid to the balance of PCE, lifetime, and cost. Otherwise, the large-scale fabrication process may not be sufficiently developed, and though the material cost is low, there are still many challenges in furthering the manufacturing process. As a next step, a future research direction could be to focus on solving the decomposition problem of perovskite in hot and humid environments and improving device stability. These issues may be solved through improvements in material engineering, including component control, interface engineering, and efficient packaging technology. In addition, the toxicity of Pb also attracts attention, since the use of Pb may hinder commercial development, but the efficiency of lead-free devices is still generally low, so research on lead-free perovskite devices is also a key issue that needs to be improved. In terms of commercialization, both the fabrication cost and the manufacturing process should be taken into account when investigating how to achieve a good-quality film with uniform coverage of large areas, along with improvements in productivity. At the same time, employing stacking technology with silicon-based solar cells may be an effective method to improve efficiency. There are still big breakthroughs to be made in the optimization of the structure of stacking devices, to ensure their long-term stability for commercialization. In terms of the manufacturing economy, the cost of raw perovskite materials is low as the amount required is small (the thickness of the film is usually only a few hundred nanometers), meaning that the cost of perovskite-based devices is significantly lower than the cost of silicon-based devices. In addition, purification and wafer cutting of silicon-based devices take place at a high temperature (>1000 °C), but perovskite devices can be fabricated at a low temperature (<150 °C). As a result, the investment cost of the related equipment is greatly reduced. Currently, although some methods are available to achieve large-scale fabrication, further improvements yet to be made in terms of good uniformity and high yield. In general, the cost of perovskite modules is much lower than that of silicon modules, but stacked modules of perovskite and silicon may push up costs, due to their complex fabrication process. In terms of environmental footprint, perovskite devices still have a long-term risk of Pb leakage, which needs to be solved through packaging and recycling technology. Meanwhile, the efficiency of lead-free perovskite devices is still low (~14%), meaning that they are still far from commercialization. In addition, the issue of solvent emission, such as the widely used DMF or DMSO, etc., can be reduced by replacement of the ionic liquid or the use of a dry process. Finally, in terms of lifecycle analysis, overall, at the manufacturing stage, the scrap ration is about 10%. Meanwhile, in the adoption phase, long-term stability is still a key factor, and the life of T_80_ has exceeded 1000 h at 85 °C/85% RH. When comparing the lifespan of perovskite-based devices with the 25-year lifespan of silicon devices, there are still many efforts that need to be made. Greater efforts must be made to explore the functional materials, structures, and novel approaches that are suitable for fabricating stable and large-area perovskite devices.

## Figures and Tables

**Table 1:** Comparison of different perovskite fabrication methods [93,94,95,96,97,98,99,100,101].

Method	Uniformity	Material Consumption	Production Efficiency	Cost	Scalability	Best PCE of PSC (%)	Best PCE of PSM (%)
Spin-coating	High	High	Slow	Low	Poor	26.7	22.5
Blade coating	Medium	Low	Medium	Low	Good	25.31	23.34
Slot-die coating	High	Medium	Fast	Medium	Excellent	23.6	18.6
Spray-coating	Medium	Low	Medium	Medium	Good	22.43	18.83
Screen-coating	Low	Low	Fast	Low	Good	20.52	18.12
Inkjet printing	Medium	High	Slow	Medium	Good	24.57	18.2

## Data Availability

No new data were created in this study.

## Supplementary Materials

The following supporting information can be downloaded at https://www.mdpi.com/article/10.3390/molecules30102221/s1: Table S1: Different perovskite fabrication methods and their corresponding performance based on a small-area (<1 cm^2^) device.
