# Supplementary material for: Strategies and Methods for Upscaling Perovskite Solar Cell Fabrication from Lab-Scale to Commercial-Area Fabrication
Source: Molecules. 2025 May 20;30(10):2221. doi: 10.3390/molecules30102221 (PMC12114390; doi:10.3390/molecules30102221)
Supplement: Supplementary file 1 [file molecules-30-02221-s001.zip › molecules-3561192-supplementary.pdf]

Supplementary Materials

Table S1 The different perovskite fabrication method and corresponding performance of small area(＜1 cm^2) device

Method	J_{sc} (mA cm^{-2})	V_{oc} (V)	FF (%)	PCE (%)	Stability (compared to initial PCE)
Spin-coating	26.49	1.193	84.5	26.7	-
Blade-coating	25.84	1.182	82.9	25.31	~100% aging 2000h (N_2, RT)
Slot-die coating	24.8	1.16	81.4	23.6	＞90% aging 1100h (RT, 20% RH)
Spray-coating	25.28	1.11	79.76	22.43	＞90% aging 1000h (indoor drying tower, RT, 30-50% RH)
Screen-coating	23.12	1.14	77.9	20.52	92.8% aging 4000h (N_2, RT)
Inkjet printing	25.37	1.18	82.1	24.57	92% aging 1041h 1 sun illumination, N_2
